# Structural Analysis of CsoS1A and the Protein Shell of the Halothiobacillus neapolitanus Carboxysome 

**DOI:** 10.1371/journal.pbio.0050144

**Published:** 2007-05-22

**Authors:** Yingssu Tsai, Michael R Sawaya, Gordon C Cannon, Fei Cai, Eric B Williams, Sabine Heinhorst, Cheryl A Kerfeld, Todd O Yeates

**Affiliations:** 1 Molecular Biology Institute, University of California Los Angeles, Los Angeles, California, United States of America; 2 Department of Chemistry and Biochemistry, University of California Los Angeles, Los Angeles, California, United States of America; 3 University of California Los Angeles–United States Department of Energy Institute for Genomics and Proteomics, Los Angeles, California, United States of America; 4 Department of Chemistry and Biochemistry, University of Southern Mississippi, Hattiesburg, Mississippi, United States of America; 5 Life Sciences Core, University of California Los Angeles, Los Angeles, California, United States of America; Stanford University, United States of America

## Abstract

The carboxysome is a bacterial organelle that functions to enhance the efficiency of CO_2_ fixation by encapsulating the enzymes ribulose bisphosphate carboxylase/oxygenase (RuBisCO) and carbonic anhydrase. The outer shell of the carboxysome is reminiscent of a viral capsid, being constructed from many copies of a few small proteins. Here we describe the structure of the shell protein CsoS1A from the chemoautotrophic bacterium Halothiobacillus neapolitanus. The CsoS1A protein forms hexameric units that pack tightly together to form a molecular layer, which is perforated by narrow pores. Sulfate ions, soaked into crystals of CsoS1A, are observed in the pores of the molecular layer, supporting the idea that the pores could be the conduit for negatively charged metabolites such as bicarbonate, which must cross the shell. The problem of diffusion across a semiporous protein shell is discussed, with the conclusion that the shell is sufficiently porous to allow adequate transport of small molecules. The molecular layer formed by CsoS1A is similar to the recently observed layers formed by cyanobacterial carboxysome shell proteins. This similarity supports the argument that the layers observed represent the natural structure of the facets of the carboxysome shell. Insights into carboxysome function are provided by comparisons of the carboxysome shell to viral capsids, and a comparison of its pores to the pores of transmembrane protein channels.

## Author Summary

Bacterial cells are generally viewed as being relatively simple because they lack the membrane-bound organelles that help organize the interiors of eukaryotic cells. However, many bacterial cells produce large, protein-based microcompartments that serve effectively as simple organelles. These microcompartments enclose specific cellular enzymes, thereby successfully sequestering particular reactions or pathways from the rest of the cytosol. The prototypical bacterial microcompartment is the carboxysome, which is found in many bacteria that fix CO_2_ into organic carbon. In these bacteria, the efficiency of CO_2_ fixation is enhanced by having the key enzymes in that pathway encapsulated together. Carboxysomes were discovered more than 40 years ago, but an understanding of their assembly and function is just beginning to emerge. Here we report new structures of the proteins that form the outer shell of the carboxysome. These structures provide further evidence that the carboxysome shell is constructed according to principles similar to those seen in icosahedral viral capsids. The structure of the carboxysome serves as a model for understanding a variety of primitive bacterial organelles that are coming to light.

## Abstract 3

The structure of the bacterial carboxysome shell protein consists of tightly packed hexameric units perforated by narrow pores that bind sulfate ions, enabling metabolites to be conducted across the shell.

## Introduction

Although bacterial cells were once thought to be relatively simple, it is becoming increasingly clear that they benefit from organized interiors [1,2]. Specialized structures have been evolved by a number of bacterial species to provide organization at the subcellular level [3–5]. Some of these structures appear to serve roles that are parallel to those served by membrane-bound organelles traditionally associated with eukaryotic cells. An organelle may be defined operationally as a structure that serves to sequester a specific set of molecules and reactions from the rest of the cell. One subcellular structure known as the carboxysome, though it is surrounded by a protein shell rather than a membrane, serves precisely that role in many bacteria [6–8]. Studies on the carboxysome and other related bacterial microcompartments promise insights into areas ranging from molecular evolution to biophysics.

**Figure 1:**
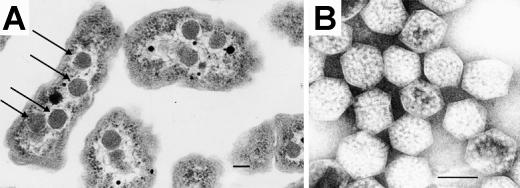
Electron Micrographs of H. neapolitanus Carboxysomes (A) A thin-section electron micrograph of H. neapolitanus cells with carboxysomes inside. In one of the cells shown, arrows highlight the visible carboxysomes. (B) A negatively stained image of intact carboxysomes isolated from H. neapolitanus. The features visualized arise from the distribution of stain around proteins forming the shell as well as around the RuBisCO molecules that fill the carboxysome interior. Scale bars indicate 100 nm.

Carboxysomes were first identified by electron microscopy in 1961 as polyhedral structures inside cyanobacteria [9]. They were subsequently identified in chemoautotrophic bacteria [10]. Although their geometrically regular appearance suggested an immediate similarity to viruses (Figure 1), carboxysomes were determined to be protein-based microcompartments for sequestering cellular enzymes for CO_2_ fixation [10]. Carboxysomes are now understood to be part of a carbon-concentrating mechanism (CCM) evolved by certain bacteria to allow for efficient CO_2_ fixation under low CO_2_ concentrations [6,11–13]. The first part of the CCM involves transmembrane protein pumps that actively transport inorganic carbon (i.e., bicarbonate or sometimes CO_2_) into the cytosol. The carboxysome represents the second part of the CCM, wherein bicarbonate is fixed into organic carbon metabolites.

Despite its discovery more that 40 years ago, the carboxysome remains rather poorly understood. Many of the details regarding how the carboxysome is constructed and how it functions are unknown. From studies on cyanobacteria and chemoautotrophs, models for carboxysome function have been developed [6,8,12,14,15]. According to current views, bicarbonate enters the carboxysome, where it is then converted to CO_2_ by carbonic anhydrase (CA). Then, the enzyme ribulose bisphosphate carboxylase/oxygenase (RuBisCO) catalyzes the reaction of CO_2_ with ribulose bisphosphate (RuBP) inside the carboxysome to produce two molecules of 3-phosphoglyceric acid (3PGA). No other Calvin cycle enzymes are known to be encapsulated, so 3PGA is presumed to leave the carboxysome. Due to the weak binding affinity of RuBisCO for CO_2_, the increased local concentrations of CO_2_ and RuBisCO inside the microcompartment allow for more efficient fixation of carbon [16,17]. Beyond the advantage gained by co-localizing RuBisCO and CA, the carboxysome could provide further advantages if its shell were selectively permeable to different small molecules, particularly because molecular oxygen competes with CO_2_ in its reaction with RuBisCO, but direct evidence for selective permeability is lacking. In organisms that have carboxysomes, nearly all of the RuBisCO in the cell is encapsulated (Figure 1B), whereas essentially none is observed in the cytosol [18,19]. How RuBisCO and CA are assembled and encapsulated within carboxysomes is not yet understood. In addition to RuBisCO, CA, and the proteins making up the shell of the carboxysome (discussed below), several other proteins have been identified as components of the carboxysome [20], but their functional roles are not yet clear.

The carboxysome shell is formed mainly by the assembly of many copies of a few small shell proteins. Even within a single bacterial species, carboxysomes exhibit some variation in size. The total number of shell subunits must therefore be variable, but it is at least a few thousand. In Halothiobacillus neapolitanus, several of the genes involved in carboxysome function, including those that code for the shell proteins, are encoded in the *cso* operon. The small shell proteins are named CsoS1, for ***c***arboxy***so***me ***s***hell ***1*** *,* and are encoded on the bacterial chromosome in the order: CsoS1C, CsoS1A, and CsoS1B. The CsoS1A and CsoS1C proteins differ by only two amino acids out of 98, whereas the CsoS1B protein has an extended C-terminus of 12 amino acids compared to CsoS1A and CsoS1C. However, if the additional amino acids in CsoS1B are not considered, the three CsoS1 paralogs are greater than 80% identical to each other in amino acid sequence [6,20,21]. The precise ratio of CsoS1A, -B, and -C proteins in the H. neapolitanus carboxysome shell is unknown. In all microbes that contain carboxysomes, multiple paralogs of the shell protein can be found. Proteins homologous to the carboxysome shell protein have been identified by sequence comparison in some 50 organisms across the bacterial kingdom [7], including microbes that do not fix CO_2_. Numerous other bacteria evidently have evolved related microcompartments that presumably encapsulate different enzymes for diverse cellular functions [14]. The best characterized of these other microcompartments are those that function in propanediol utilization *(pdu)* and ethanolamine utilization *(eut)* [22]. The *pdu* and *eut* microcompartments have been characterized in *Salmonella* [23,24]. The *eut* shell is also present in Escherichia coli.

Carboxysomes are classified into two types, alpha and beta, based on sequence homology and gene organization [17]. The carboxysomes of chemoautotrophic bacteria and some marine cyanobacteria are of the alpha type, whereas most cyanobacterial carboxysomes are of the beta type. The nature and extent of the structural and functional differences between the two kinds of carboxysomes are not understood yet [17]. The first structures of carboxysome shell proteins were determined recently from *Synechocystis* PCC 6803 (*Syn.* 6803), a cyanobacterium containing beta carboxysomes [7]. Here the crystal structure is reported for CsoS1A. This protein and the nearly identical CsoS1C protein are the main constituents of the alpha carboxysome shell in the chemoautotroph H. neapolitanus. Insights into structural and mechanistic principles are provided by comparisons to the structures of the beta carboxysome shell proteins [7], and to other proteins that form shells or that contain pores for transport.

## Results

### Structure of CsoS1A, a Carboxysome Shell Protein from H. neapolitanus

**Figure 2:**
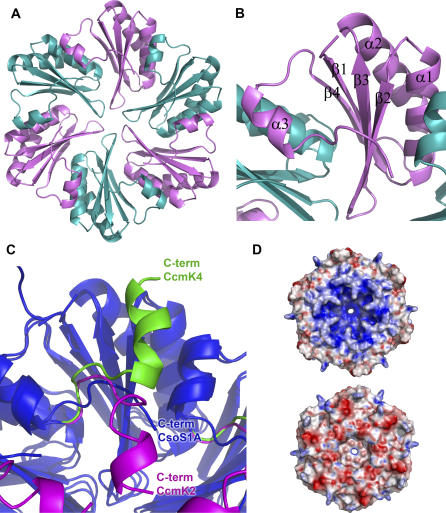
Structure of the CsoS1A Hexamer That Forms the Building Block of the Alpha Type Carboxysome Shell (A) A ribbon diagram with the monomeric units colored alternately in purple and teal. (B) A close-up of the CsoS1A monomer in the hexamer. Secondary structures are labeled and numbered. (C) An illustration of the differing C-terminal configurations of carboxysome shell proteins: CsoS1A (blue), CcmK2 (pink), and CcmK4 (green). The differences between the structures are apparent after residue 91, with the C-terminus of CsoS1A intermediate in position compared to the termini of CcmK2 and CcmK4. (D) An illustration of the geometric and electrostatic differences between the concave (top) and convex (bottom) sides of the CsoS1A hexamer. The concave side of CsoS1A is composed of mostly positive electrostatic potential (blue), whereas the convex side is composed of mostly negative electrostatic potential (red).

The structure of CsoS1A was determined at a resolution of 1.4 Å (Figure 2A and 2B), revealing an α/β fold highly similar to that observed previously for the beta carboxysome shell subunits CcmK2 and CcmK4 from the cyanobacterium *Syn.* 6803 [7]. The C-terminal ends of these three shell subunits differ in length; CsoS1A is 15 residues shorter than CcmK4 and eight residues shorter than CcmK2. Excluding seven residues at the C-terminal end of the protein, CsoS1A can be overlapped onto CcmK2 and CcmK4 with root mean square (rms) deviations of only 0.7 Å over the C-alpha backbone positions. However, the CsoS1A protein diverges in structure from the others in the C-terminal region, after residue 91 (Figure 2C). In the C-terminal regions, the differences between CsoS1A and the other shell proteins are around 8 Å on average. CcmK2 and CcmK4 also differ structurally from each other at the C-terminus. This difference appears to affect the differential assembly of those proteins into higher order structures [7]. In CsoS1A, the C-terminal region occupies a position intermediate to that observed in CcmK2 and CcmK4, and is disposed to allow sheet formation, as described below.

CsoS1A forms a cyclic hexameric building block from six monomeric subunits, as observed previously in the CcmK2 and CcmK4 shell subunits from *Syn*. 6803 [7]. The CsoS1A hexamer is very similar to those described earlier. With the exception of the C-terminal extensions noted above, the CsoS1A hexamer can be superimposed on the CcmK2 hexamer with an rms deviation of only 0.9 Å over the C-alpha backbone positions. The hexamer has a central pore. The pore is short because the hexamer is thin in its central region. This situation arises from a major depression at the center of the hexamer on one of its faces, and a minor depression on the other side. As a result, the two sides of the hexamer have different shapes, with one side appearing to be strongly concave and the other being more nearly convex (Figure 2D).

### Structure of the Molecular Layer

**Figure 3:**
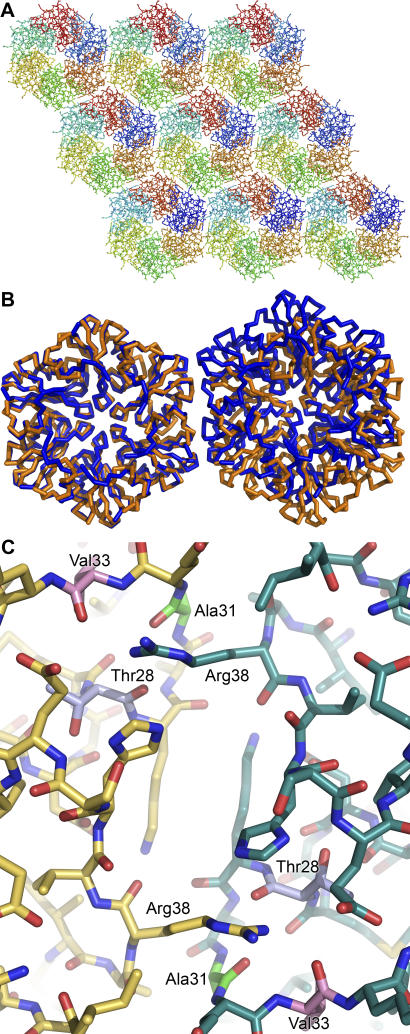
Packing of CsoS1A Hexamers in a Layer (A) Individual CsoS1A molecules are colored differently in each hexamer. CsoS1A molecules in the same hexameric position are colored the same. (B) Backbone alignment of adjacent CsoS1A hexamers and adjacent CcmK2 hexamers [7] from the beta-type cyanobacterial carboxysome shell. CsoS1A is indicated in blue, and CcmK2 is shown in orange. The shift between adjacent hexamers in CsoS1A compared to CcmK2 leads to the tighter packing. (C) A close-up view of the interaction between two adjacent CsoS1A hexamers. The two hexamers are colored separately in yellow and gray. Arg83 from each hexamer is shown surrounded by residues belonging to the adjacent hexamer: Val33 (pink), Ala31 (green), and Thr28 (light blue).

In the CsoS1A crystal structure, each hexamer fits together tightly with the complementary edges of other hexamers to form a flat molecular layer (Figure 3A). This is the third instance in which carboxysome hexamers have been seen to pack into molecular layers [7]. The similarity in packing of shell subunit hexamers from both alpha and beta carboxysomes in multiple crystal forms further supports the argument that the relatively flat facets of the natural carboxysome shell are comprised of sheets of hexamers.

A close inspection of the CsoS1A layer reveals a difference in hexamer packing compared to those reported previously. Although the alpha and beta carboxysome hexamers are highly similar to each other when examined in isolation, the precise relationship between adjacent hexamers within the layer differs. In particular, there is an approximately 8-Å shift at the interface where two hexamers meet (Figure 3B). This results in a somewhat tighter packing of hexamers in CsoS1A. The tight packing between CsoS1A hexamers is stabilized by three intermolecular hydrogen bonds between the guanidinium moiety of Arg83 and the carbonyl oxygen atoms of Thr28, Ala31, and Val33 at the C-terminal end of helix 1 (Figure 3C). Furthermore, the positive charge of the arginine complements the negative dipole of helix 1 in the neighboring hexamer. In addition, tight packing is affected through highly complementary van der Waals surfaces. The shape complementary between two adjacent CsoS1A hexamers is 0.72, which is in the range for protein–protein inhibitor interfaces [25]. In CcmK2, the shape complementary is 0.63, which is similar to those observed in antibody–antigen interfaces. In addition, the distance between the centers of two adjacent hexamers is 66.4 Å for CsoS1A, compared to 69.7 Å for CcmK2.

Although the molecular layer has a thickness that varies at different positions, it is possible to evaluate an average thickness of the shell. This can be done objectively by taking into account the repeating unit of the layer, the mass of the protein that occupies the repeating unit, and the density of typical proteins (see Materials and Methods). The average thickness of the CsoS1A layer is 18.6 Å, which, given the roughly 1,000-Å diameter of the carboxysome, is quite thin relative to the capsids of large viruses. The structures of a wide variety of icosahedral viral capsids were examined, including examples from 24 different types (genera) (Figure S2, Table S1). There are a number of small viruses whose capsids are as thin as the carboxysome shell. For example, the satellite panicum mosaic virus shell [26] is 15.8-Å thick on average, but its average diameter is only 159 Å. Most viruses, particularly those that are larger (e.g., having triangulation numbers greater than three), tend to have thicker capsids. Bacteriophage HK97 provides a lone counterexample. That viral capsid is only 14.7-Å thick on average despite its large size (diameter of 587 Å). The unusually thin viral shell in HK97 is rendered stable by a unique arrangement of covalent bonds between protein chains, which leads to interlinked rings of subunits [27]. By comparison to more typical viral capsids, the carboxysome shell is unusually thin, especially in view of its large size.

An understanding of the spacing between hexamers can also be used to estimate the number of protein subunits in the carboxysome shell. This value is approximately 4,800 subunits for a 1,000-Å diameter carboxysome (see Materials and Methods). If carboxysomes are constructed according to principles similar to those used by triangulated icosahedral viral capsids [28], this would correspond to a triangulation number near 80 (i.e., 4,800/60), with *T* = 81 being the closest allowable triangulation number for a triangulated icosahedron. A recent electron microscopy study provides new evidence that carboxysomes are roughly icosahedral [29], but whether they conform strictly to principles of quasi-equivalence and icosahedral triangulation is not yet known.

### Sidedness of the Layer

One side of the carboxysome shell faces outward towards the cytosol, while the other faces inward, presumably interacting with the enzymatic constituents of the carboxysome. In the molecular layers we have visualized, it is not known yet which side faces inward and which faces outward. Although this will have to be established experimentally, a comparison of the two sides offers some clues. An uneven distribution of charged amino acid residues gives rise to an electrostatic difference between the two sides (Figure 2D). The concave side of the hexamer shows a strong positive electrostatic potential, whereas the other side is slightly negative. One might expect to see a relationship between the charge on the inner side of the carboxysome and the charges on the other components of the carboxysome (i.e., the other enzymes and proteins contained within or potentially attached to the shell). Most of these proteins, including the major constituents (RuBisCO and CA), would carry a net negative charge at the pH of the H. neapolitanus cytosol (a pH of approximately 7.8 [30]) (Table S2). This finding tends to implicate the concave side of the hexamer, which is positively charged, as the inward facing side of the layer.

**Figure 4:**
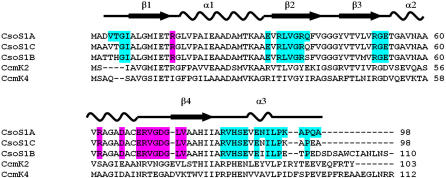
Alignment of the Amino Acid Sequence of H. neapolitanus CsoS1A to Its Paralogs and to the Two *Syn.* 6803 Orthologs of Known Structure Accessible residues on the concave side are in cyan, whereas those on the convex side are in magenta. Accessible residues that are conserved between all three CsoS1 paralogs are also colored in the other species.

Sequence comparisons provide another potential source of inference. The inner surface of the shell most likely interacts with one or more other proteins inside the carboxysome. One might expect such interactions to constrain the sequence divergence of amino acids on the inward facing side. To make an evaluation possible, amino acid positions constituting the two surfaces were identified. In total, 11 amino acids (from each monomer) were judged to be exposed on the convex side, whereas 29 were exposed on the concave side. Figure 4 illustrates the accessible amino acid residues in a multiple sequence alignment. The three homologous CsoS1 proteins from H. neapolitanus were compared to ascertain the degree to which the accessible amino acids are conserved in sequence. On the concave side, 22 out of 29 amino acids (76%) are perfectly conserved between the three protein sequences, whereas all 11 accessible residues (100%) are conserved on the convex side. Over the entire protein, 92 residues out of 98 (94%) are conserved. For a second comparison, 20 proteins in the known sequence database identified as being most similar to CsoS1A were aligned. The trend was the same in this wider comparison. On the concave side, 16 of the 29 accessible residues (55%) are conserved, whereas all 11 (100%) remain conserved on the convex side (see Figure S1). The high sequence conservation on the convex side of the hexamer suggests that it might be the inward-facing side. But this implication contradicts the one based on the electrostatics argument above. In order to determine which of these lines of reasoning is correct and which is faulty, the sidedness of the carboxysome shell will have to be determined experimentally.

### Characteristics of the CsoS1A Pore

The pore at the center of the CsoS1A hexamer is approximately 4 Å across at its narrowest point. A similarly narrow pore was seen in the structure of the CcmK4 hexamer from *Syn.* 6803; the CcmK2 hexamer had a wider pore, approximately 7 Å across [7]. The CsoS1A pore is different in certain respects from both of those seen earlier. In the shell proteins from *Syn.* 6803, the pore is lined by positively charged residues (Lys30 in CcmK2, and Arg38 in CcmK4). The corresponding position in CsoS1A is a phenylalanine (Phe40). Nonetheless, though the electrostatic potential in the CsoS1A pore is not as extreme, it retains a positive potential (owing to other charged residues such as Arg34, Arg38, and Arg51).

**Figure 5:**
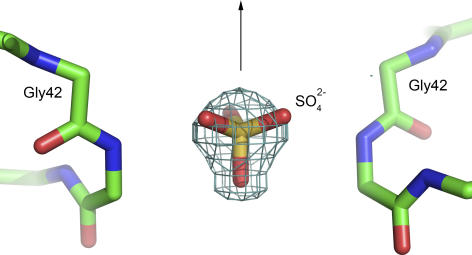
A Sulfate Ion Found in the Pore of the CsoS1A Hexamer The (Fobs-Fcalc) difference electron density map shown was calculated using diffraction data from a crystal soaked in sodium sulfate. The map is contoured at 3σ. The view is with the pore (arrow) running vertically in the plane of the paper. The pore is lined by the polypeptide backbone from all six proteins in a hexamer (two copies are shown). The identity of the sulfate ion was supported by an anomalous difference map (Figure S3).

The possibility that the pores seen in the carboxysome shell hexamers might serve for transport has motivated attempts to visualize metabolites in the pore by X-ray crystallography. However, attempts to achieve binding of bicarbonate, 3PGA, or RuBP in the pore were previously unsuccessful using crystals of the CcmK proteins. They were similarly unsuccessful here using crystals of CsoS1A, despite the pore being evidently large enough and electrostatically complementary. However, it was possible to visualize bound sulfate ions after soaking a crystal in 200 mM sodium sulfate. Sulfate can be considered an analog for bicarbonate, having a similar size and a charge of the same sign, though twice as large in magnitude. Difference electron density maps (Figure 5) suggested possible sulfate ions at three locations. One of these sites was in the hexagonal pore, whereas the others were at the locations where the 2-fold and 3-fold axes of symmetry pass through the layer, i.e., where two hexamers meet at an edge and where three meet at a corner. Because electron density peaks on symmetry axes can sometimes be spurious, additional analysis was performed. Sulfur atoms scatter X-rays anomalously, so an anomalous difference Fourier map was used to visualize possible sulfate ions (Figure S3). This calculation confirmed the sulfate ion in the hexagonal pore, but was not definitive with regard to the other putative sulfate binding sites. In the hexagonal pore, the sulfate is in contact with Gly43, which is part of a Gly-Gly-Gly motif that is conserved across the three CsoS1 proteins (A, B, and C) and other closely related shell proteins, but not in the CcmK proteins. The negatively charged sulfate oxygen atoms are poised to make hydrogen bonds to the backbone amide nitrogen of Gly43, and possibly to the C-alpha hydrogen atom of that residue; the potential importance of hydrogen bonds involving C-alpha hydrogen atoms has been discussed, but their strength remains an open question [31,32].

**Figure 6:**
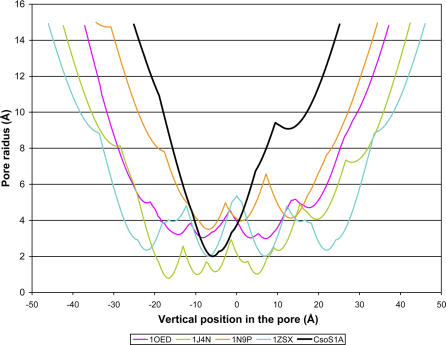
Calculated Radius of the Central Pore through CsoS1A, Shown in Comparison to the Pores through Transmembrane Channel Proteins In each case, the radius of the pore is plotted as a function of the vertical position in the layer. CsoS1A is indicated in black and compared to the acetylcholine receptor pore (1OED), the aquaporin1 water channel (1J4N), the cytoplasmic domain of the inward rectifier potassium channel 1 (1N9P), and the human potassium channel Kv β-subunit (1ZSX). The CsoS1A pore is much shorter in length and more conical in shape than the pores through transmembrane channel proteins. The position of the CsoS1A curve along the horizontal axis was chosen arbitrarily.

A comparison can be made between the hexameric pores of the carboxysome and the pores through oligomeric membrane protein channels. One notable difference is the thinness of the carboxysome hexamer at the narrowest point of the pore. The CsoS1A hexamer pore was compared to the pores in four transmembrane channels: acetylcholine receptor pore (1OED) [33], aquaporin1 water channel (1J4N) [34], the cytoplasmic domain of the inward rectifier potassium channel 1 (1N9P) [35], and human potassium channel Kv β-subunit (1ZSX) (Figure 6). In the transmembrane proteins examined, the central pore ranges in length from approximately 30 Å to 40 Å, corresponding roughly to the thickness of a lipid bilayer. In contrast, the pore through the carboxysome has a thickness of only about 10 Å, beyond which the pore quickly expands. The shape of the carboxysome pore is therefore much more conical (on either side) compared to the pores of transmembrane channels, which remain narrow and more cylindrical along their lengths.

## Discussion

The structure of CsoS1A, a major component of the alpha carboxysome shell, provides crucial support for emerging ideas about the structural basis of carboxysome function. It is particularly significant that the main observations here are in agreement with those of our earlier study on the beta carboxysome (CcmK) shell proteins [7]. The repeated observation of a conserved hexagonal layer structure provides a compelling argument that this represents the biological structure of the facets of the shell. The conserved tightness of the hexagonal protein packing reinforces the idea that the carboxysome shell probably serves not only to co-localize certain enzymes, but likely also plays an important role in controlling what is able to enter and exit the carboxysome. The tightness of the packing can be contrasted to that seen in protein cages formed by S-layer proteins on the outer surface of numerous species of microbes [36–38]. S-layers serve primarily for structural integrity rather than transport control, and this is reflected in the sizes of the openings created when those proteins pack into layers: S-layer pore diameters range from 25 Å to 45 Å [39,40].

Despite the similarities in the packing of shell proteins, some differences are also notable. In particular, the packing of the CsoS1A hexamers is somewhat tighter than that observed in the recent CcmK studies, as a result of a 8-Å lateral shift between hexamers. One potential explanation is that the looser packing in the earlier structures might have reflected a slight disruption under crystallization conditions. However, the evidence argues against this. In the earlier study on the CcmK proteins from *Syn*. 6803, layer structures were observed for two different proteins, CcmK2 and CcmK4. The side-to-side packing of hexamers in those two cases was nearly identical, with spacings of 70.5 Å and 69.7 Å, respectively. The near equivalence of the spacing in those two layers is consistent with the idea that multiple subunit types within a single organism must be able to pack together in a commensurate fashion in a single carboxysome. If that idea is correct, then structures of the other CsoS1 proteins (B and C) will be expected to pack with a spacing of approximately 66 Å, as in CsoS1A. Whether the observed difference in packing tightness reflects a categorical difference between alpha and beta carboxysomes remains to be seen. Another possible explanation would be that the porosity of a layer could be controlled by such differences in packing. If this were the case, then it might be possible to visualize the same shell subunit in two different packing configurations. This has not been observed so far.

The visualization of sulfate ions inside the pores of the CsoS1A layer indicates that transport of small molecules such as bicarbonate is probably feasible. If no other enzymatic activities aside from RuBisCO and CA can be attributed to the carboxysome, then the three-carbon and five-carbon metabolites, 3PGA and RuBP, would also have to cross the shell. These are larger than bicarbonate, but not dramatically larger in cross-section. It is possible that protein flexibility could permit their transfer through the pores. Another possibility is that other protein components not yet identified might provide transport of three-carbon and five-carbon metabolites across the shell. Whether the pores identified here and in the CcmK proteins truly serve as pores for metabolite transport has not yet been established by direct experiments, but the current structural knowledge now makes possible mutagenesis experiments aimed at answering those questions.

Notable differences are seen between the pores through the carboxysome shell and those seen in transmembrane channels. The pores through transmembrane channels tend to be relatively long, remaining narrow over most of the distance across the membrane. In contrast, carboxysome pores have a more conical shape on either side of a central constriction (Figure 6). This difference probably reflects a combination of functional and structural factors. The lengths of transmembrane pores are dictated to a large degree by the lipid bilayer. The tendency of transmembrane pores to remain narrow over a relatively large distance may also relate to their generally high selectivity for the ions or small molecules they transport. In contrast, the carboxysome shell is thinner than a lipid bilayer, and the restricted region of the pore is relatively short. This observation may be an indication of relatively low selectivity by the carboxysome pore. As noted above, general electrostatics likely play a role, but this may be the extent of the selectivity. Recall that sulfate (carrying a −2 charge) could be seen bound in the pore, whereas the natural intermediate bicarbonate was not seen to be tightly bound. Tight binding of bicarbonate within the pore would presumably be counterproductive. Experimental approaches for addressing the permeability of microcompartment shells have not been developed yet.

The close molecular packing in the carboxysome shell raises a question as to whether this might critically limit the diffusive flux of intermediates such as bicarbonate from the cytosol; if so, any other advantages conferred by sequestering RuBisCO and CA within the shell could be negated. The pores in the carboxysome shell occupy only a very small fraction of the surface. Assuming that the flux of intermediates across the shell is a critical issue, one might intuitively think that a dramatically greater flux would be made possible by a significant increase in the size of the pores, or a decrease in the spacing between them. However, this intuitive notion is not correct. Owing to the peculiarities of molecular diffusion, efficient capture of diffusing molecules is achieved at a very low density of receptors or pores on the surface of a cell (or the carboxysome). That argument has been articulated by Berg et al. with regard to diffusion to cell surface receptors [41,42]. By a similar analysis (see Materials and Methods), we argue that, though the pores occupy only a small fraction of the surface, the carboxysome shell probably does not present a severe obstacle to the net flux of small molecules such as bicarbonate from the cytosol to the carboxysome interior. The calculations suggest that the pores in the carboxysome shell are spaced sufficiently close together to create a relatively high degree of porosity.

The architecture of the carboxysome shell suggests comparisons to viral capsids. A survey of known structures shows that the protein shell of the carboxysome is somewhat thinner than the shells of typical viral capsids. This may reflect differences between the functional roles of the two kinds of shells. Viral capsids must remain stable under potentially destabilizing conditions outside the host cell. In addition, they may experience internal pressure from the tightly packed nucleic acid molecules they encapsulate [43,44]. In carboxysomes and other related bacterial microcompartments, the emphasis may be on diffusion efficiency, which would be more favorable across a thinner shell with shorter pores.

Despite the generally greater thickness of viral capsids, there are strong similarities between viral capsids and bacterial microcompartments, as was noted before [7]. There is no direct evidence that any virus evolved from bacterial microcompartments, or vice versa. Nonetheless, there are important evolutionary implications. For example, the question of how cells could have originated during evolution is confounded by a chicken-and-egg paradox: the synthesis of proteins relies on the nucleic acids that encode them, whereas the replication of nucleic acids relies on proteins for synthesis. Viruses serve as a minimalistic paradigm for this paradox, with the nucleic acid component relying on the protein shell for protection, and the proteins relying on the nucleic acid for encoding. Such paradoxes provide a difficult challenge for Darwinian evolution, whose principles call for the acquisition of complex properties in a stepwise fashion. The problem is in understanding how selective advantages might have been gained by scenarios lying partway along the evolutionary paths to complex cellular systems. The carboxysome provides a compelling illustration of how a protein shell (aside from any encapsulated genetic material) could confer a selective advantage. In principle, subcellular protein shells of various types could represent intermediate forms in the origin of viruses.

### Conclusion

Though the carboxysome and other related microcompartments are still only poorly understood, some of their mechanistic features are beginning to come to light. The comparison between carboxysome pores and transmembrane protein channels provides some early insights into molecular transport across the carboxysome shell. With preliminary structural models in hand, computational studies should be able to shed more light on how energetics, kinetics, and molecular diffusion underlie the ability of the carboxysome to enhance CO_2_ fixation. The structures also provide the basis for future site-directed mutagenesis studies aimed at dissecting function at the atomic level. Finally, the architectural similarity between bacterial microcompartment shells and viral capsids provides fertile ground for considering questions of molecular and cellular evolution.

## Materials and Methods

### DNA cloning and protein expression.

The expression clone pCsoS1A-ProEx was generated by first amplifying the *CsoS1A* gene from pTn1 [45] using Pfu Turbo DNA polymerase (Stratagene, http://www.stratagene.com) with forward primer 1AfBamHI (5′-CGAGGATCCATGGCTGATGTAACTGG-3′) and reverse primer 1ArSacI (5′-GGTCGAGCTCGGAATATCTGACTTAGG-3′). Forward and reverse primers contain engineered restriction sites (underlined in the primer sequence) that allow for in-frame ligation into the multicloning region of the prokaryotic expression vector pProEx-HTb (Life Technologies, http://invitrogen.com). The QIAquick (Qiagen Sciences, http://www1.qiagen.com) column-purified PCR gene product and the expression vector were each digested sequentially with BamHI then SacI at 37 °C for 1.5 h each. The PCR product and vector were each re-purified between sequential digests by QIAquick column purification. The double-digested vector and insert were ligated with T4 DNA ligase at 16 °C for 16 h with a 5:1 insert to vector ratio. The expression vector was transformed into DH5α competent E. coli and verified by sequencing recovered plasmid DNA. The overexpression and purification of CsoS1A was accomplished as described previously for the ProEx-based recombinant protein purification system [46]. AcTEV Protease (Invitrogen, http://www.invitrogen.com) was used to cleave at the Tobacco Etch Virus (TEV) cleavage site to remove the N-terminal histidine tag.

### Crystallography and analysis.

The crystallization condition for CsoS1A was 30% (v/v) PEG 400 and 0.1 M CHES (pH 9.5) from Wizard Screen II (Emerald Biosystems, http://www.emeraldbiosystems.com). Crystals formed within 2 mo. Diffraction data were collected to 1.8-Å resolution on Raxis-IV++ at 1.54-Å wavelength at the University of California, Los Angeles, and to 1.4-Å resolution at the Advanced Light Source (ALS) in Berkeley, California, at 1.0-Å wavelength. Processing of data was performed using DENZO and SCALEPACK [47]. Molecular replacement was performed for the 1.8 Å data using as a search model the structure of CcmK4 with the program PHASER [48]. The 1.8 Å and 1.4 Å data were isomorphous, so refinement of the molecular replacement model against data to 1.4-Å resolution was straightforward. Refinement and model building were performed with Refmac [49] and O [50]. Data collection statistics are given in Table S3.

A soak of CsoS1A crystals in sodium sulfate was performed in a solution of 30% (v/v) PEG 400, 0.1 M CHES (pH 9.5), and 0.2 M Na_2_SO_4_. Diffraction data were collected at ALS to a resolution of 1.6 Å. Processing of data was performed using DENZO and SCALEPACK [47]. The structure was solved using difference Fourier methods with the 1.8-Å structure of CsoS1A. Refinement was performed using Refmac [49] and Coot [51].

Various structure calculations, including surface complementarity and surface accessibility, were performed using CCP4 [52]. Protein characteristic calculations were performed using ProtParam [53]. The electrostatic potential was calculated using the Ezprot package of programs [54]. Refinement statistics are listed in Table S3.

### Calculation of CsoS1A layer thickness.

The thickness of the CsoS1A protein layer was calculated by dividing the calculated volume of one protein hexamer by the unique area it occupies in the molecular layer. The total mass of the hexamer was calculated by multiplying the mass of one CsoS1A protein (9,831.3 g/6.02 · 10^23^) by six. The volume was calculated by dividing this mass by the density of a typical protein, 1.35 g/cm^3^. The area occupied by a single hexameric unit is (67 Å)^2^3^1/2^/2. The thickness was then calculated by dividing the volume by the area to give an average thickness of 18.6 Å.

### Calculation of the number of subunits in the shell.

If the carboxysome is approximated as a sphere of radius 500 Å, its surface area would be 4π(500 Å)^2^. As noted above, six protein subunits occupy an area of (67 Å)^2^3^1/2^/2. The number of subunits in the shell of a typical carboxysome is therefore approximately 6 · 4π(500 Å)^2^/((67 Å)^2^3^1/2^/2), or approximately 4,800.

### Calculation of channel sizes.

Pore sizes were calculated using the program HOLE [55]. The axis of the pore was extracted by observing the graphical image of the PDB file. The membrane channels utilized for comparison were acetylcholine receptor pore (1OED), aquaporin1 water channel (1J4N), cytoplasmic domain of the inward rectifier potassium channel 1 (1N9P), and human potassium channel Kv β-subunit (1ZSX).

### Calculation of virus capsid thickness.

The virus shell diameters were obtained from VIPERdb [56]. The average diameter was used to calculate the average thickness of the shell. The mean value between the minimum and maximum diameter reported was taken as an approximation for the average diameter. The thickness of a capsid was calculated by finding the total mass by calculating the number of subunits in one triangular facet of the icosahedral shell (three times the triangulation number *T*) and multiplying the number of subunits by the mass of a single protein or protein complex. The volume was determined by dividing the mass by the density of protein, 1.35 g/cm^3^. The area of the face was calculated by dividing the total surface area (4π*r* ^2^, where *r* is the radius of the virus shell) by the number of identical faces in an icosahedral capsid, which is 20. The thickness was then determined by dividing the volume by the area. The 42 viral capsid proteins calculated were from 24 different genera (Table S1).

### Analysis of diffusion across a semiporous layer.

The problem of diffusion to a set of small adsorbing patches or holes in a large sphere has been analyzed before in the context of diffusion of molecules in solution to receptors on a cell surface [42]. A similar treatment applies here for the diffusion of molecules from the cytosol to holes in the surface of the carboxysome. The degree to which small holes would limit flux has been quantified by comparing (1) the rate that a set of small adsorbing patches would capture diffusing molecules to (2) the rate of capture that would be achieved if the entire surface was adsorbing (i.e., as if the entire surface constituted an open pore). This unitless ratio is a measure of the efficiency of transport that can be achieved by some given density of receptors or pores.

The problem can be treated analytically by solving the equations of diffusion under steady-state conditions [41]. The rate of capture for the entire spherical surface (given a unit concentration difference between infinity and the spherical surface) is 4π*Da,* where *D* is the diffusion coefficient of the molecule and *a* is the radius of the large sphere (the carboxysome in the present case). The rate of capture for the same sphere, but where only *N* circular patches of radius *s* are able to capture, is 4π*Da*/(1 + π*a*/*Ns*), which is similar to the first expression except for division by the factor 1 + π*a*/*Ns*. The relative efficiency of transfer—the ratio between the rates of capture in the second case compared to the first—is therefore simply 1/(1 + π*a*/*Ns*). This expression for capture efficiency shows that the efficiency approaches unity asymptotically as the number of patches increases or as their size increases, as expected. What is surprising, however, is the extremely low density and small patch size that is sufficient to achieve good efficiency. The values of *a, N,* and *s* for the carboxysome are illustrative. The sizes of carboxysomes vary somewhat, but here we take the radius *a* to be 500 Å. The density of holes in the carboxysome is dictated by the spacing of the hexameric units. That value is nearly conserved at approximately 67 Å in the layer structures we have determined (this study and [7]). This is the spacing between the hexagonal pores described in detail above. As noted, there could also be other locations in the layer where movement of ions would be allowed, but here we consider only the prominent hexagonal pores. Treating the carboxysome as a sphere and assigning an area of (67 Å)^2^3^1/2^/2 for the repeating surface unit, the value for the number of pores *(N)* would be approximately 800. If the radius of the CsoS1A pore is taken to be 2 Å (its value at the narrowest point), the relative capture efficiency 1/(1 + π*a*/*Ns*) has a calculated value of approximately 50%. Larger pores with a radius of approximately 3 Å were seen in the CcmK2 structure. The calculated efficiency in that case is 75%. According to this analysis, the holes in the carboxysome shell are spaced sufficiently close to allow relatively efficient diffusion to the pores.

The issue of movement *through* the pores is not considered here in detail, but continuing with a steady-state diffusion treatment, it can be shown that the resistance for diffusing through the pores is small compared to the resistance for diffusing *to* the pores when the length of the pores (taken to be cylindrical) remains smaller than π*s*. The relative shortness of the carboxysome pore (i.e., the thinness of the shell at the site of the pore) was discussed above. According to Figure 4, the CsoS1A pore remains narrower than 3 Å in radius over a length of only about 6 Å, which is less than π · 3 Å.

The treatment above is, of course, only a first approximation, subject to numerous caveats. A more detailed analysis involving modeling and numerical simulations will be required to gain further insights. In particular, the energetic and geometrical properties of the pore will have to be considered in detail, especially with regard to the effects electrostatics might have on the relative flux of charged versus uncharged molecules, such as bicarbonate and molecular oxygen.

## Supporting Information

Figure S1 Alignment of CsoS1A with the First 20 Homologous Proteins Identified in a Search of the Non-Redundant Sequence Database, Using BLASTp with Default Settings The GenBank GI numbers are given for the top hits. Accessible residues on the concave side of the hexamer are in cyan, whereas those on the convex side are in magenta.
(36 KB PPT)

Figure S2 Plot of the Calculated Average Thickness of Viral Capsid Protein Shells as a Function of Their Average Diameters The carboxysome shell (CsoS1A) is indicated in pink, whereas the capsid proteins are indicated in blue. Viral capsids that are approximately as thin as the carboxysome layer are mainly of a much smaller diameter (200 to 400 Å).
(267 KB TIF)

Figure S3 Electron Density Maps from a Crystal Soaked in Sulfate Showing a High-Density Peak on the Symmetry Axis of the Hexagonal Pore (A) In addition to the Fobs-Fcalc difference map in the text (Figure 5), (A) shows an anomalous difference Fourier map with high density at the position of the sulfur atom due to its anomalous X-ray scattering. The peak is 6.1 standard deviations above the mean.
(B) An (Fobs_sulfate_ − Fobs_native_), φ_model_, difference map also shows density for a bound sulfate ion.
(382 KB JPG)

Table S1 Icosahedral Virus Capsids Used in the Calculation of Shell Thickness (68 KB DOC)

Table S2 Approximate Net Charges for Proteins Associated with the Carboxysome (38 KB DOC)

Table S3 Data Collection and Refinement Statistics (46 KB DOC)

### Accession Numbers

The coordinates and structure factors of native and sulfate-bound CsoS1A have been entered in the Protein Data Bank (http://www.rcsb.org/pdb) under the accession numbers 2EWH and 2G13, respectively.

## Abbreviations

3PGA: 3-phosphoglyceric acid
CA: carbonic anhydrase
CCM: carbon-concentrating mechanism
RuBisCO: ribulose bisphosphate carboxylase/oxygenase
