# Supplementary material for: Structural Analysis of CsoS1A and the Protein Shell of the Halothiobacillus neapolitanus Carboxysome
Source: PLoS Biol. 2007 May 22;5(6):e144. doi: 10.1371/journal.pbio.0050144 (PMC1872035; doi:10.1371/journal.pbio.0050144)
Supplement: Supplementary file 1 [file pbio.0050144.st001.doc]

| Table S1. Icosahedral virus capsids used in the calculation of shell thickness.^a^ | | | | |
| --- | --- | --- | --- | --- |
| PDB ID | Average diameter (Å) | Average thickness (Å) | Family | Genus |
| 1STM | 159 | 15.78 | Satellites | Sattelite |
| 1JS9 | 261 | 21.12 | Bromoviridae | Bromovirus |
| 1FRS | 262 | 14.11 | Leviviridae | Levivirus |
| 1GAV | 263 | 13.89 | Leviviridae | Levivirus |
| 2MS2 | 263 | 14.00 | Leviviridae | Levivirus |
| 1NY7 | 274 | 20.32 | Comoviridae | Comovirus |
| 1DWN | 276 | 12.86 | Leviviridae | Levivirus |
| 1PGL | 276 | 18.94 | Comoviridae | Comovirus |
| 1A6C | 277 | 17.45 | Comoviridae | Nepovirus |
| 1AUY | 279 | 18.25 | Tymoviridae | Tymovirus |
| 1M06 | 281 | 20.50 | Microviridae | Microvirus |
| 1LAJ | 281 | 21.54 | Bromoviridae | Cucumovirus |
| 1F15 | 281 | 21.58 | Bromoviridae | Cucumovirus |
| 1E57 | 283 | 17.60 | Tymoviridae | Tymovirus |
| 1DDL | 283 | 17.62 | Tymoviridae | Tymovirus |
| 1C8N | 285 | 26.03 | Tombusviridae | Necrovirus |
| 1F2N | 286 | 22.42 | Sobemovirus | Sobemovirus |
| 1NG0 | 288 | 23.39 | Sobemovirus | Sobemovirus |
| 1ZBE | 291 | 20.04 | Picornaviridae | Apthovirus |
| 1BEV | 297 | 22.70 | Picornaviridae | Enterovirus |
| 1OOP | 297 | 22.98 | Picornaviridae | Enterovirus |
| 1COV | 297 | 23.03 | Picornaviridae | Enterovirus |
| 1MQT | 298 | 22.74 | Picornaviridae | Enterovirus |
| 1H8T | 298 | 23.21 | Picornaviridae | Enterovirus |
| 2MEV | 299 | 22.22 | Picornaviridae | Cardiovirus |
| 1AYM | 299 | 23.08 | Picornaviridae | Rhinovirus |
| 1TME | 300 | 22.39 | Picornaviridae | Cardiovirus |
| 1RHI | 300 | 22.74 | Picornaviridae | Rhinovirus |
| 1D4M | 300 | 23.26 | Picornaviridae | Enterovirus |
| 1B35 | 306 | 22.45 | Dicistroviridae | Cripavirus |
| 1QGT | 312 | 16.29 | Hepadnaviridae | Orthohepadnavirus |
| 1F8V | 312 | 30.91 | Nodaviridae | Alphanodavirus |
| 1NOV | 315 | 30.64 | Nodaviridae | Alphanodavirus |
| 2BBV | 315 | 31.19 | Nodaviridae | Alphanodavirus |
| 1OPO | 322 | 25.72 | Tombusviridae | Carmovirus |
| 1CD3 | 330 | 14.56 | Microviridae | Microvirus |
| 1OHF | 399 | 41.26 | Tetraviridae | Omegatetravirus |
| 1M1C | 403 | 22.01 | Totiviridae | Totivirus |
| 1OHG | 587 | 14.70 | Siphoviridae | Lambda-like |
| 1WCE | 659 | 33.21 | Birnaviridae | Avibirnavirus |
| 2BTV | 669 | 26.31 | Reoviridae | Orbivirus |
| 1UF2 | 695 | 29.42 | Reoviridae | Phytoreovirus |

^1^

^a^The viruses included are icosahedral and follow the principle of quasi-equivalence.
