# Supplementary material for: Structural Analysis of CsoS1A and the Protein Shell of the Halothiobacillus neapolitanus Carboxysome
Source: PLoS Biol. 2007 May 22;5(6):e144. doi: 10.1371/journal.pbio.0050144 (PMC1872035; doi:10.1371/journal.pbio.0050144)
Supplement: Supplementary file 2 [file pbio.0050144.st002.doc]

| Table S2. Approximate net charges for proteins associated with the carboxyxome^a^ | | | |
| --- | --- | --- | --- |
| *H. neapolitanus* |  | *Syn.* PCC 6803 |  |
| CsoS1A | -2 | CcmK1 | -1 |
| CsoS1B | -6 | CcmK2 | -3 |
| CsoS1C | -2 | CcmK3 | -1 |
| OrfA | -2 | CcmK4 | -1 |
| OrfB | -1 | CcmO | -6 |
| CbbL | -10 | CcmL | +2 |
| CbbS | -1 | CcmM | +7 |
| CsoS2 | +17 | CcmN | -11 |
| CsoSCA^b^ | -8 | RbcL | -10 |
|  |  | RbcS | -3 |
|  |  | Slr1347^c^  ^2^ | -9 |
|  |  |  |  |
| Spinach |  |  |  |
| RuBisCo L | -7 | CA | -9 |
| RuBisCo S | -2 |  |  |

^a^ Net charges were calculated by subtracting the number of negatively charged residues (Asp and Glu) from the number of positively charged residues (Arg and Lys).

^b^ CsoSCA is the carboxysomal carbonic anhydrase in *H. neapolitanus.*

^c^ Slr1347 is a carbonic anhydrase believed to be associated with the *Syn*. 6803 carboxysome [1].

Reference:

1. So AK-C, Espie GS (1998) Cloning, characterization and expression of carbonic anhydrase from the cyanobacterium *Synechocystis* PCC6803. Plant Mol Biol 37: 205–215.
