# Supplementary material for: Structural Analysis of CsoS1A and the Protein Shell of the Halothiobacillus neapolitanus Carboxysome
Source: PLoS Biol. 2007 May 22;5(6):e144. doi: 10.1371/journal.pbio.0050144 (PMC1872035; doi:10.1371/journal.pbio.0050144)
Supplement: Supplementary file 3 [file pbio.0050144.st003.doc]

| Table S3. Data collection and refinement statistics | | |
| --- | --- | --- |
| Ligand | None | SO_4_ |
| Metal | None | None |
| *Data collection statistics* | | |
| Resolution (Å) | 57.54 - 1.40 | 57.35 - 1.61 |
| Space group | P6 | P6 |
| Unit cell dimensions | | |
| a (Å) | 66.417 | 66.196 |
| b (Å) | 66.417 | 66.196 |
| c (Å) | 28.982 | 27.421 |
| Radiation source | ALS 8.2.2 | ALS 8.2.2 |
| Radiation wavelength (Å) | 1.0 | 1.0 |
| Measured reflections | 114978 | 130257 |
| Unique reflections | 13639 | 130257 |
| Overall completeness (%) | 93.8 | 99.8 |
| Overall completeness (I/σ>0) (%) | 84.9 | 99.03 |
| Last shell completeness (%) | 71.8 | 98.5 |
| Overall R_sym_^a^ | 0.073 | 0.101 |
| Last shell R_sym_ | 0.395 | 0.562 |
| Overall I/σ (I) | 24.8 | 23.1 |
| Last shell I/σ (I) | 2.42 | 4.88 |
| *Refinement statistics* | | |
| R_work_^b^ | 0.185 | 0.218 |
| R_free_^c^ | 0.24 | 0.26 |
| rmsd bond lengths (Å) | 0.017 | 0.012 |
| rmsd bond angles (degrees) | 1.691 | 1.613 |
| Overall B-factor | 28.56 | 15.95 |
| Solvent atoms | 52 | 31 |
| Average solvent B-factor | 42.53 | 24.97 |
| PDB ID code | 2EWH | 2G13 |

^a^R_sym_(I) = Σ_hkl_( (Σ_i_|I_hkl,i_ - <I_hkl_>|) / Σ_i_I_hkl,i_)

^b^R_work_ = Σ_hkl_ |F_obs_ - F_calc_| / Σ_hkl_ F_obs_

^c^R_free_ = Σ_hkl_ |F_obs_ - F_calc_| / Σ_hkl_ F_obs_, where all reflections belong to a test set of 5% randomly selected data.
